# Protective effect of enterovirus-71 (EV71) virus-like particle vaccine against lethal EV71 infection in a neonatal mouse model

**DOI:** 10.3892/mmr.2015.3680

**Published:** 2015-04-24

**Authors:** LEI CAO, FENGFENG MAO, ZHENG PANG, YAO YI, FENG QIU, RUIGUANG TIAN, QINGLING MENG, ZHIYUAN JIA, SHENGLI BI

**Affiliations:** 1 Department of Viral Hepatitis, National Institute for Viral Disease Control and Prevention, China Center for Disease Control and Prevention, Beijing 102206, P.R. China; 2 Laboratory Animal Center, The Fourth Military Medical University, Xi’an, Shaanxi 710032, P.R. China

**Keywords:** enterovirus-71, virus-like particle, vaccine, model

## Abstract

Enterovirus-71 (EV71) is a viral pathogen that causes severe cases of hand, foot and mouth disease (HFMD) among young children, with significant mortality. Effective vaccines against HFMD are urgently required. Several EV71 virus-like particle (VLP) vaccine candidates were found to be protective in the neonatal mouse EV71 challenge model. However, to what extent the VLP vaccine protects susceptible organs against EV71 infection *in vivo* has remained elusive. In the present study, the comprehensive immunogenicity of a potential EV71 vaccine candidate based on VLPs was evaluated in a neonatal mouse model. Despite lower levels of neutralizing antibodies to EV71 in the sera of VLP-immunized mice compared with those in mice vaccinated with inactivated EV71, the VLP-based vaccine was shown to be able to induce immunoglobulin (Ig)G and IgA memory-associated cellular immune responses to EV71. Of note, the EV71 VLP vaccine candidate was capable of inhibiting viral proliferation in cardiac muscle, skeletal muscle, lung and intestine of immunized mice and provided effective protection against the pathological damage caused by viral attack. In particular, the VLP vaccine was able to inhibit the transportation of EV71 from the central nervous system to the muscle tissue and greatly protected muscle tissue from infection, along with recovery from the viral infection. This led to nearly 100% immunoprotective efficacy, enabling neonatal mice delivered by VLP-immunized female adult mice to survive and grow with good health. The present study provided valuable additional knowledge of the specific protective efficacy of the EV71 VLP vaccine *in vivo*, which also indicated that it is a promising potential candidate for being developed into an EV71 vaccine.

## Introduction

Enterovirus 71 (EV71) is a member of the *Picornaviridae* family of the enterovirus genus and is one of the main etiological agents responsible for hand-foot-mouth disease (HFMD) in humans (1,2). EV71 is a non-enveloped virus with a single-stranded RNA genome consisting of P1, P2 and P3 regions (3). The P1 protein is further cleaved into VP1, VP3 and VP0 by protease 3CD, while the other two regions encode seven proteins responsible for replication and virulence (3). VP1, VP3 and VP0 can spontaneously co-assemble into the icosahedral empty procapsid (2). A portion of VP0 can be autocleaved to yield VP2 and VP4, which are associated with infectious EV71 virions (4). EV71 infections can cause more severe neurological complications than other enteroviruses and can lead to high morbidity rates in children (5,6). Since its initial identification in 1969, several HFMD epidemics have occurred worldwide, particularly in Asia-Pacific regions (7,8). In China, outbreaks of EV71 infection have been reported throughout the country with increasing prevalence, particularly during the last 10 years (9). Several investigations have focused on the prevention of EV71 infections, and numerous approaches have been tested to develop a safe and effective EV71 vaccine (10,11).

Virus-like particles (VLPs) have attracted increasing attention as great potential vaccine candidates, as they are non-infectious particles consisting of all the major structural proteins, mimicking the organization and conformations of the native particle; however, they are devoid of viral nucleic acids and are non-infectious (12). VLP-based prophylactic vaccines have been successful against hepatitis B virus and human papillomavirus and are now commercially available. Recombinant EV71 VLPs have been shown to be neutralization antibodies and confer a degree of protection from EV71 infection in a neonatal mouse model (13–15).

Variable virus proliferation has been demonstrated in the central nervous system and associated organs during EV71 infections (16,17). Therefore, the generation of immunoprotective responses in infected animals and indicators of pathological responses from the protection by the EV71 vaccine also require an objective assessment. However, to what extent the VLP vaccine protects susceptible organs against EV71 infection *in vivo* remains elusive. Preliminary studies have indicated that neutralizing anti bodies induced by VLPs may be able to efficiently neutralize the homologous live EV71 virus and a panel of two C4 strains isolated in China (data not shown). In the present study, the efficacy of an EV71 vaccine candidate based on VLPs was evaluated; furthermore, the significance and value of assessing the immunogenicity and immunoprotection of vaccine candidates in ICR mice were further elucidated by using a range of methods, including pathological, etiological and lethal challenge analyses.

## Materials and methods

### Viruses and VLP vaccine preparation

The human EV71 FY-15 strain (C4 genogroup, isolated in Fu Yang, Anhui, China, 2008) was used for immunization. Another highly mouse-adapted virulent EV71 strain (C4 genogroup) supplied by the National Vaccine and Serum Institute (Beijing, China) was used in the challenge experiments. The two EV71 viruses were propagated in rhabdomyosarcoma (RD) cells using minimum essential medium (MEM; Gibco-BRL, Invitrogen Life Technologies, Grand Island, NY, USA) supplemented with 2% fetal bovine erum (FBS; Gibco-BRL). For virus purification, the FY-15 virus was precipitated with 7% polyethylene glycol 8000 (Amresco, LLC, Solon, OH, USA) and 2% NaCl (Sinopharm Chemical Reagent Co., Ltd., Beijing, China) and then centrifuged (110,000 ×g, 3 h) over 15% cesium chloride (CsCl; Sinopharm Chemical Reagent Co., Ltd.). Virus pellets were re-suspended in PBS (pH 7.4), sonicated for 30 sec and centrifuged (10,0000 ×g, 20 h) over a continuous CsCl gradient (10–40%). The resultant virus bands were dialyzed against phosphate-buffered saline (PBS). Purified FY-15 virus was examined by eryo-electron microscopy (Cryo-EM; Tecnai Tf20; FEI, Houston, TX, USA) and inactivated with 1/4,000 formalin (Sigma-Aldrich, St Louis, MO, USA) for 96 h at 37°C (18). Another mouse-adapted EV71 virus was harvested and centrifuged (5,000 ×g, 10 min) to remove debris. The viral titer was evaluated using RD cells by microtitration assay and expressed as the 50% tissue culture-infective dose (TCID_50_) (19).

EV71 VLPs were produced in serum-free medium as previously described (20). Briefly, Sf9 cells (Invitrogen Life Technologies) were infected with a recombinant baculovirus (AcMNPV-P1-3CD; constructed previously in the Department of Viral Hepatitis) for co-expression of P1 and 3CD derived from the EV71 FY-15 strain at a multiplicity of infection (MOI) of 1 in 1l CellSTACK-10 culture chambers (Corning-Costar, Corning, NY, USA) at 27°C for 96 h. The infected cells were harvested with a Tris-NaCl (Invitrogen Life Technologies) buffer containing 1% NP-40 (Sigma-Aldrich) and cell debris was removed by centrifugation (9,000 ×g, 20 min). Then the supernatant was centrifuged over 15% CsCl and a discontinuous sucrose gradient (10–60%). Purified VLPs were examined by transmission electron microscopy (TEM; Tecnai 12; FEI). The protein concentrations were quantified using a Nanodrop (Thermo Fisher Scientific, Waltham, MA, USA). A solution of the inactivated virus and VLPs each was then mixed with adjuvant Al(OH)_3_ (2 mg/ml; Sinovac Biotech Co., Ltd, Beijing, China) at a volumetric ratio of 1:1 for *in vivo* studies.

### Immunization of adult mice and sampling procedures

Three groups (n=10/group) of 4-5 week-old female ICR mice (Beijing HFK Bioscience Co., Ltd., Beijing, China) were immunized by intramuscular injection with 5 *μ*g (100 *μ*l/mouse) of either purified VLPs, inactivated whole virus or PBS. The mice were subjected to a 12 h dark/12 h light cycle, with *ad libitum* access to food and water. The mice were housed 5 mice/cage in a room maintained at 19–24°C with 47–63% relative humidity. Animal treatment and care were provided in accordance with the guidelines of the National Institute for Viral Disease Control and Prevention, China Center for Disease Control and Prevention (Beijing, China). All procedures used in the present study were approved by the Animal Care and Welfare Committee at the National Institute for Viral Disease Control and Prevention, China Center for Disease Control and Prevention. The immunization schedule consisted of three inoculations administered at two-week intervals. Blood was sampled from the tail at weeks 0, 2, 4, 6, 8, 10 and 14 for monitoring the immune responses. Mice (3 or 4 per group) were sacrificed at week 6 and 10 for measurement of the cellular immune responses. Lung wash, vaginal wash and small intestine samples were collected at week 6 in order to test secretory immunoglobulin (Ig)A levels. All these samples were immediately placed on ice and stored with 100 mM phenylmethyl sulfonyl fluoride (Sigma-Aldrich) at −20°C until further analysis. The remaining mice in each group were used to breed neonatal mice for lethal virus challenge experiments at week eight (week four post-boost) and sacrificed at week 14.

### Measurement of EV71-specific antibody

Anti-EV71 IgG and IgG subclasses in serum as well as IgA in mucosal secretions were measured by ELISA. In brief, plates coated with inactivated native virus (5 *μ*g/ml) were blocked (PBS containing 0.05% Tween 20 (Sinopharm Chemical Reagent Co., Ltd.) and 5% bovine serum albumin (Amresco, LLC) and washed prior to the addition of the two-fold serially diluted serum samples (1:2^2^–22^2^) and 1:2-diluted mucosal secretion samples were added. Plates were incubated for 1 h and washed prior to addition of 0.15 *μ*g/ml horseradish-peroxidase (HRP)-conjugated goat anti-mouse IgG (1:30,000 dilution; cat. no. A9917; Sigma-Aldrich) or 0.125 *μ*g/ml goat anti-mouse IgA (HRP) secondary antibody (1:8,000 dilution; cat. no. ab97235; Abcam, Cambridge, MA, USA) was added. The anti-EV71 IgG isotypes in mouse sera were determined using a mouse antibody isotyping kit (ISO 2; Sigma-Aldrich) according to the manufacturer’s instructions. After 30 minutes, color development was initiated by adding 100 *μ*l tetramethylbenzidine substrate (Sigma-Aldrich) and terminated by adding 50 *μ*l of H_2_SO_4_ (2M; Sinopharm Chemical Reagent Co., Ltd.). The optical density at 40 nm (OD_450_) was read using an ELISA reader (FI-01621; Thermo Scientific Multiskan™ GO; Thermo Fisher Scientific Oy, Vantaa, Finland).

### Neutralizing antibody assays

Microneutralization assays were performed against EV71 in RD cells. Briefly, 50 *μ*l two-fold diluted serum was mixed with 50 *μ*l 100 TCID_50_ EV71 in 96-well plates. RD cells were added after 1 h and incubated for 3–4 days at 37°C. The neutralizing titer was determined as the highest dilution that gave no cellular cytopathic effects (CPE).

### Enzyme-linked immunospot (ELISpot) assays. interleukin

(IL)-4-, interferon (IFN)-γ- and IgA-secreting splenocytes were measured using ELISpot for mouse IL-4, IFN-γ (U-Cytech, Utrecht, the Netherlands) and IgA kits (Mabtech AB, Nacka Strand, Sweden). Briefly, for detection of virus-specific IL-4 and IFN-γ, 96-well microplates (Millipore, Billerica, MA, USA) were pre-coated with anti-IL-4 or anti-IFN-γ monoclonal antibody (mAb) and incubated overnight at 4°C. Plates were blocked with RPMI-1640/10% FBS for 1 h at room temperature, followed by addition of splenocytes (3×10^5^/well). Subsequently, 250 *μ*g/ml inactivated EV71 virus was added (20 *μ*l/well) to stimulate effector cells. Phytohaemagglutinin (PHA; 9 *μ*g/well, 37°C, 16 h; Gibco-BRL) stimulation was used for the positive control. For IgA ELISpot, microplates were first coated overnight with 10 *μ*g/ml anti-IgA antibody or 30 *μ*g/ml inactivated EV71 virions and blocked with PBS-10% FBS for 30 min. Then splenocytes (1×10^5^/well) were added. After incubation of the splenocytes for 16–18 h, following removal of the cells, plates were processed according to the manufacturer’s instructions. Spot-forming cells (SFC) were counted using an automated ELISpot reader (Cellular Technology Ltd, Shaker Heights, OH, USA).

### Lethal viral challenges in neonatal mice

Four weeks after the final immunization, female adult mice were allowed to mate. The neonatal mice were challenged intracerebrally with the EV71 virus attack strain [10^6^ PFUs (plaque-forming units)/ml, 10 *μ*l/mouse] within 72 h following birth. Mice were monitored for mortality daily over 14 days. In addition, three neonatal mice from each group were used for histopathological analysis at five days after EV71 challenge. The neonatal mice were sacrificed using isoflurane (Sigma-Aldrich). Extracted organs were fixed in 4% paraformaldehyde for 24 h and processed for paraffin embedding. Tissues sections (5 *μ*m) were deparaffinized using xylene (Sinopharm Chemical Reagent Co., Ltd.) and hydrated with a graded series of alcohol (Sinopharm Chemical Reagent Co., Ltd.). Sections were stained with hematoxylin and eosin (Sigma-Aldrich), for morphological evaluation. Immunohistochemical analyses were performed as described previously (21). Briefly, hydrated sections were treated with 0.25% trypsin (Invitrogen Life Technologies) solution containing 0.5% CaCl_2_ (Sinopharm Chemical Reagent Co., Ltd.) in PBS for 30 min and incubated with 3% H_2_O_2_ (Sinopharm Chemical Reagent Co., Ltd,) in methanol to block endogenous peroxidase activity, followed by incubation with PBS containing 5% BSA. The treated sections were then incubated with anti-EV71 mAb (1:300 dilution; cat, no. 05-0001; AbMax Biotechnology Co., Ltd., Beijing, China) at 4°C overnight. The sections were washed three times with PBS and then incubated with HRP-conjugated goat anti-mouse IgG (1:1,000 dilution; cat. no. ZB-2305; ZSGB-BIO, Beijing, China) for 1 h at 37°C. The sections were visualized using 3-3′diaminobenzi-dine (10 min) and hematoxylin (30 sec), then examined using a light microscope (IX51; Olympus Corporation, Tokyo, Japan).

### Statistical analysis

All data obtained were processed and analyzed using SPSS software (version 16.0; SPSS, Inc., Chicago, IL, USA). Figures were plotted and analyzed using GraphPad Prism software (GraphPad, La Jolla, CA, USA). P<0.05 was considered to indicate a statistically significant difference between values.

## Results

### Characterization of purified VLPs

**Figure 1:**
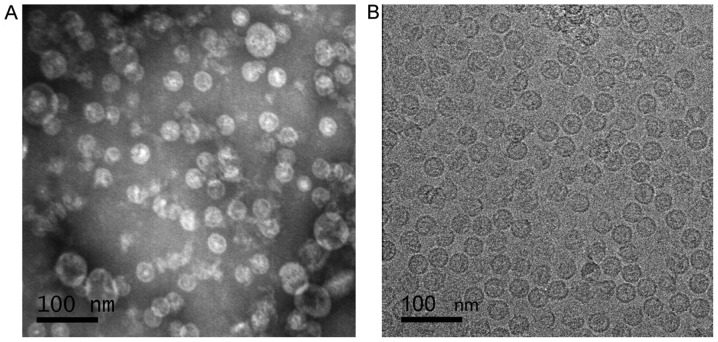
Characterization of purified VLPs. (A) Transmission electron microscopy image of VLPs produced by recombinant baculovirus (AcMNPV-P1-3CD)-infected Sf9 cells co-expressing EV71 P1 and 3CD. VLPs were purified by ultracentrifugation. (B) EV71 virions after ultracentrifugation were characterized by cryo-electron microscopy. Scale bar, 100 nm. EV, enterovirus; VLP, virus-like particle.

Purified VLPs and EV71 virus were visualized by TEM and Cryo-EM. Purified VLPs (Fig. 1A) exhibited icosahedral morphology similar to those of EV71 virions (Fig. 1B) and the size was ~25–27 nm. These EV71 VLPs were used as a vaccine in the subsequent experiments.

### Humoral responses elicited by EV71 VLP immunization

**Figure 2:**
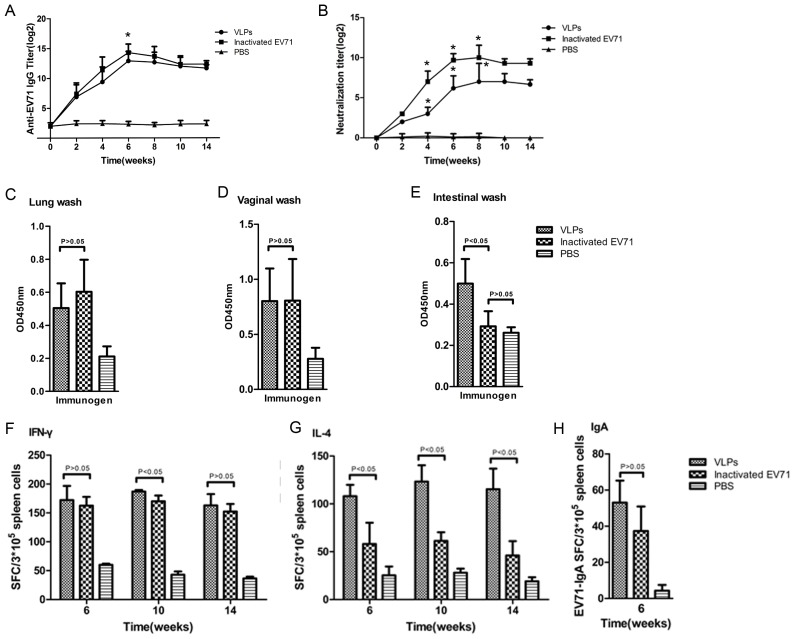
Titer profiles of humoral and cellular immune responses to EV71 in mouse models. The geometric mean titers (log2) of (A) total IgG, (B) neutralizing antibody to EV71 and (C-E) anti-EV71 secretory IgA secreted at mucosal surfaces were determined using ELISA (for total anti-EV71 IgG titration, the positive cut-off OD value was defined as 2.1 times that of normal mouse serum) and neutralization techniques. Enzyme-linked immunospot detection of specific responses of (F) IFN-γ-secreting T cells, (G) IL-4-secreting T cells or (H) IgA-secreting spleen cells to EV71 in spleen lymphocytes from immunized mice. All values are expressed as the mean ± standard deviation from 3 or 4 mice per group. EV, enterovirus; VLP, virus-like particle; SFC, spot-forming cells, PBS, phosphate-buffered saline; OD_450_, optical density at 450 nm; IFN, interferon; Ig, immunoglobulin.

Neutralization titration of sera of the mice showed that compared with levels in the control group, high levels of anti-EV71 IgG antibodies in the serum of mice were present at 2 weeks post-boost immunization with purified VLPs, reaching a maximum of 2^12^ (Fig. 2A). Compared with the VLPs, inactivated EV71 induced slightly higher levels of anti-EV71 antibodies with a significant difference at week six (P<0.05). In the two groups, the time-dependent changes in antibody levels and magnitudes of the response were similar. Peak levels of antibodies following the two types of antigen immunization were detected at week 6 and maintained for at least 14 weeks. The inactivated EV71-immunized group exhibited higher levels of all IgG subtypes (Table I); however, high levels of IgG1 and IgG2b and low levels of IgG2a and IgG3 were also detected in the serum of the VLP-immunized group, indicating a mixed Th1/Th2 response. The capacity of antibodies generated by VLP immunization to neutralize the homologous live EV71 virus was investigated (Fig. 2B). In VLP-immunized mice, significantly increased neutralization titers were observed post-boost compared with those pre-vaccination (week 4-8) (P<0.05); however, these were lower than those in the formalin-inactivated EV71-immunized mice (P<0.05). While the neutralization titer profile for the group immunized with VLPs was similar to that for the group immunized with inactivated EV71, neutralization titers in the two groups were greatest at week 8 and persisted for at least 14 weeks.

The specific IgA levels in the lung, vaginal and intestinal secretions from the vaccinated mice after the third immunization with EV71 VLPs showed a significant difference from those in the PBS group (P<0.05; Fig. 2C–E). However, the specific IgA responses in the intestinal samples from inactivated EV71-immunized mice were not significantly different from those in the PBS group, which may be due to incomplete homogenization processing, complex composition and proteases in the intestinal tract.

### Cellular immune responses elicited by VLP immunization

Compared with splenocytes from mice immunized with inactivated EV71, splenocytes from VLP-immunized mice produced the same levels of EV71-specific IFN-γ-producing T-cells (P>0.05, Fig. 2F) and higher levels of IL-4-producing T-cells in response to EV71 antigens (P<0.05; Fig. 2G), confirming the successful elicitation of Th1 and Th2 cellular immune responses. The EV71-specific IgA-secreting splenocytes were also quantified at week 6. Increased numbers of IgA-memory cells in mouse splenocytes in the VLP group and the inactivated EV71 group were found following immunization compared with those in the PBS control group (Fig. 2H; P>0.05). These results suggested that vaccination with EV71 VLPs leads to the generation of IgA-memory splenocytes with the capacity to recognize EV71 virions.

### Challenge with EV71 results in mortality of neonatal mice, which can be prevented by vaccination of their mothers

**Figure 3:**
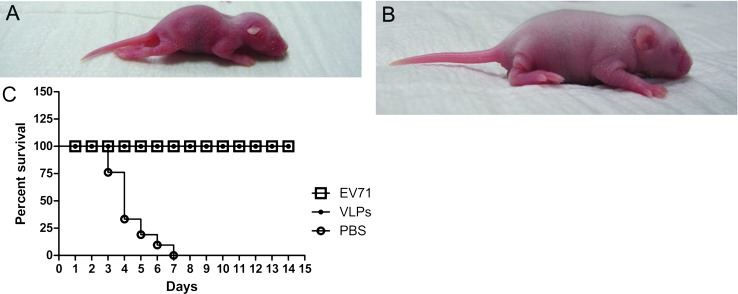
Viral infection of neonatal ICR mice with a lethal EV71 dose (10^6^ plaque-forming units per mouse). Representative images showing symptoms of (A) unimmunized and (B) VLP-immunized mice after intracerebral challenge at day three post-infection. Symptoms included weight loss, hunched back, reduced motility, hind limb paralysis and eventual mortality of unimmunized mice. VLP-immunized mice survived. (C) Survival of immunized and unimmunized mice after intranasal challenge with lethal doses of EV71. (n=20 for each group). EV, enterovirus; VLP, virus-like particle; PBS, phosphate-buffered saline.

To assess the efficacy of passive protection against the homologous EV71, neonatal ICR mice were challenged intracerebrally with EV71. As the result, nearly all inoculated mice from the PBS control group displayed a range of clinical symptoms (weight loss, hunched back, reduced motility, ruffled coat and hind limb paralysis) over the course of infection followed by death from day three (Fig. 3A and C). By contrast, all of the neonatal mice delivered by VLP-immunized and inactivated EV71 immunized female adult mice were able to survive, grew normally and were of good health, and the immunoprotective efficacy of the vaccines reached nearly 100% (Fig. 3B). These results demonstrated that VLP immunization conferred passive protection that was passed on from the female parental mice to the neonatal mice.

### Histopathological analysis shows that vaccination protects major organs of neonatal mice challenged with EV71

**Figure 4:**
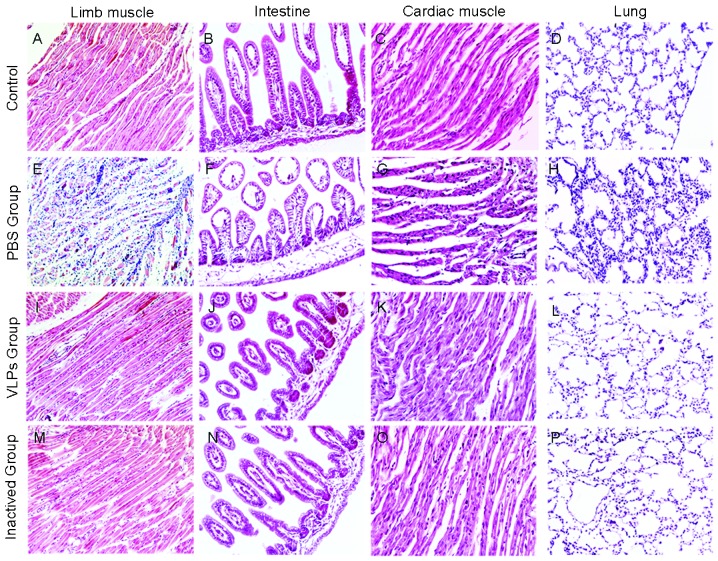
Pathological changes in various tissues of mice infected with enterovirus 71 by intracerebral inoculation. Histopathological examinations were performed on 5-*μ*m sections of paraffin-embedded tissues or organs stained with hematoxylin and eosin. Magnification, ×200. VLP, virus-like particle; PBS, phosphate-buffered saline.

The pathological changes in the brain and other major organs of immunized and non-immunized control neonatal mice were examined in parallel (Fig. 4). Compared with normal control mice, certain pathological changes were present in the PBS group (Fig. 4A–H). The hind limb muscle was most severely affected by severe diffuse necrotizing myositis and minor lymphocytic infiltration, which is likely to be responsible for the observed hind limb paralysis (Fig. 4E). The intestines revealed dilation of the villi and mild villous blunting with associated crypt hyperplasia, suggesting substantial tissue damage in the small intestines (Fig. 4F). A pathological response comprising minor myocardial interstitial edema and occasional scattered mononuclear cells invading cardiac muscles were also observed (Fig. 4G). Prominent pathological manifestations of pulmonary inflammation in lung tissue included a widened alveolar wall and extensive inflammatory cell aggregation (Fig. 4H). By contrast, under the protection of the VLP vaccines, only mild muscle atrophy was observed in the limb musculature (Fig. 4I and M). No other pathological changes were noted.(Fig. 4J–L and N–P). In addition, the limb musculature of the neonatal mice immunized with VLPs or inactivated EV71 displayed slight myofiber regeneration within nuclear rowing, suggesting signs of recovery from the viral infection.

**Figure 5:**
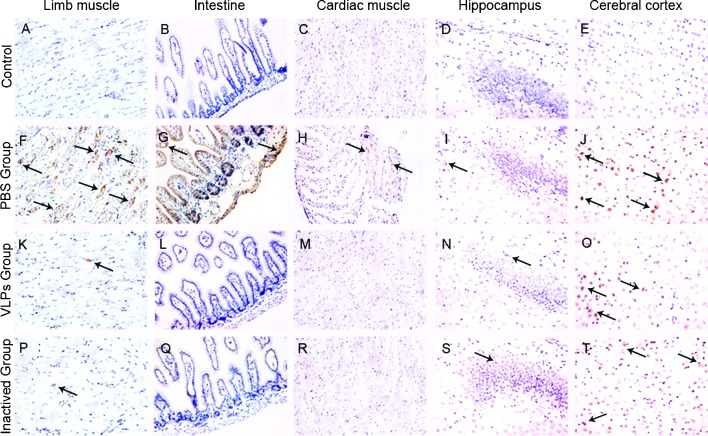
Immunohistochemical analysis of various tissues from mice infected with EV71 by intracerebral inoculation. Sections (5 *μ*m) of paraffin-embedded tissues or organs were prepared and EV71 was detected with mouse anti-EV71 monoclonal antibody and visualized with diaminobenzidine substrate. Arrows indicate positivity for viral anitigens; magnification, ×200. EV, enterovirus; VLP, virus-like particle; PBS, phosphate-buffered saline.

Further immunohistochemical analyses were in accordance with the results of the pathological analysis (Fig. 5). EV71 infiltration was mainly observed in the hind limb muscles and to a certain extent in the small intestines and cardiac muscles in the PBS group (Fig. 5F–H). Low amounts of EV71 were detected in the hippocampus, while a high level of EV71 infiltration was detected in the cerebral cortex in all groups, which may be due to the intracerebral injection of the EV71 virus attack strain in the lethal EV71 challenge experiment (Fig. 5I and J). Following vaccine-mediated immunization, only very low levels of EV71 antigen were detected in the hind limb muscles (Fig. 5K–M and P–R). All these observations demonstrated the marked immunoprotective efficacy of EV71 VLP immunization against EV71 infection.

## Discussion

Preliminary studies have indicated that neutralizing antibodies induced by VLPs were able to efficiently neutralize the homologous live EV71 virus and a panel of two C4 strains isolated in China (data not shown). Although VLPs induced similar levels of anti-EV71 IgG compared with inactivated EV71, the neutralization titers in the former case were lower. VP1 is a major antigenic epitope and has an important role in neutralizing the EV71 virus (22,23). According to studies by in Lin *et al* (11) and Wang *et al* (24), residues 210–220 of VP1, which form part of an important neutralizing epitope of EV71, lie on the capsid surface, alongside the VP2 EF loop (residues 136–150), to form a single epitope. Disruption in the order of residues 211–217 upon particle expansion may account for the reduced immunogenicity (11,24). The VP1 in the VLPs used in the present study was hypothesized to be similar with the VP1 in the empty EV71 virus particles (sedimentation, 80S; 60 copies of VP0, VP1 and VP3) cultivated and separated by Wang *et al* (24). Slight conformational changes of VP1 in empty particles may affect their neutralizing capacity.

Although antibody responses are critical for protection against EV71 infection, certain studies suggested that cellular immune responses correlate with the clinical severity of HFMD and have an important role in human immunity (13,15). Chang *et al* (13) suggested that lower EV71-specific cellular responses may be associated with immunopathogenesis of EV71-associated pulmonary edema. The different Th1 and Th2 immune response profiles corresponded to the activation of two distinct major sub-sets of T cells characterized by their pattern of cytokine production (25). The present study indicated that VLP vaccine immunization generated good IFN-γ responses and even better IL-4 responses to the EV71 antigen in mice. The ELISpot assay further demonstrated the presence of EV71-specific IgG- and IgA-secreting cells in spleens of the VLP-vaccinated mice, but not of the PBS-immunized mice. The IgG subtype profile showed that the EV71 VLP vaccine induced high levels of IgG1 and IgG2b, thus confirming the generation of a mixed Th1 and Th2 response. These results were consistent with those obtained following influenza virus and human papilloma virus VLP-immunization (26,27). The production of IgA-memory spleen cells detected following VLP vaccine immunization further confirmed successful priming by VLPs.

Protection against viral infectivity in animal models is regarded as the optimal test of vaccine efficacy (23). EV71 was presumed to infect the central nervous system (CNS) via peripheral nerves in muscle tissue followed by active retrograde axonal transport to the CNS (28). It was also reported that muscle cells were the main site of proliferation of the EV71 virus strain found around Fuyang in China (29,30). Additional studies demonstrated that skeletal muscle was the main tissue supporting EV71 replication in mice. In addition to CNS injury, necrotic myositis may also be responsible for the paralysis and death observed in EV71-infected mice (31). Thus, protection against further infection of the CNS or skeletal muscle by preventing EV71 from attacking muscle tissue and neurons is particularly important. Based on the abovementioned studies, the present study performed further pathological and etiological analyses on different organs to understand the specific passive immunoprotective effects *in vivo*. It was found that the VLP vaccine was able to inhibit the transport of EV71 from the CNS to muscle tissue and prevent infection of muscle tissue along with recovery from the viral infection. The prevention of pathological injury by effective resistance to EV71 infection in intestine and cardiac muscle tissues mediated by VLP vaccine immunization is also of critical importance. This passive protection offered great immunoprotective efficacy from the VLP or inactivated EV71 vaccines, reaching nearly 100%. Of note, the results indicated that VLP vaccines not only prevented the virus from causing serious damage to the body, but also promoted a quick recovery of the body from minor injuries resulting from EV71 infection.

It is likely that these results not only confirmed the immu-noprotective effect of the experimental VLP vaccine against EV71, but also provided valuable additional insight to aid in further exploring the detailed mechanism of action of the vaccine, and supported the protective role for the VLP vaccine in resistance and resolution following EV71 infection.

## Figures and Tables

**Table I:** IgG subtype profile in mouse sera^a^.

Type of antigen	OD_450_ of ELISA for EV71 specific IgG at serum dilution of 1:100			
	IgG1	IgG2a	IgG2b	IgG3
VLPs (n=10)	1.644 (0.111)	0.370 (0.059)	0.884 (0.042)	0.248 (0.085)
Inactivated EV71 (n=10)	1.770 (0.154)	0.958 (0.082)	1.620 (0.106)	0.759 (0.054)
PBS (n=10)	0.111 (0.011)	0.123 (0.014)	0.114 (0.004)	0.138 (0.022)

a Three groups of mice were immunized with EV71 VLPs, inactivated EV71 or PBS. Six weeks after the first immunization, mouse sera were collected and assayed for specific anti-EV71 IgG subtypes by ELISA. Serum samples of each vaccinated group were pooled and tested in triplicate in three separate experiments. The relative titer is expressed as the OD_450_ with the standard deviation shown in parentheses. EV, enterovirus; VLP, virus-like particle; PBS, phosphate-buffered saline; OD_450_, optical density at 450 nm; IgG, immunoglobulin G.
